# Effects of the intramyocardial implantation of stromal vascular fraction in patients with chronic ischemic cardiomyopathy

**DOI:** 10.1186/s12967-016-0918-5

**Published:** 2016-06-02

**Authors:** K. Comella, J. Parcero, H. Bansal, J. Perez, J. Lopez, A. Agrawal, T. Ichim

**Affiliations:** US Stem Cell, Inc, Sunrise, FL USA; Regenerative Medicine Institute, Tijuana, Mexico; Consultant Regenerative Medicine, Mother Cell Spinal Injury and Stem Cell Research, Anupam Hospital, Rudrapur, Uttarakhand 263153 India

**Keywords:** Stromal vascular fraction (SVF), Adipose derived stromal/stem cells (ADSCs), Stem cells, Adipose tissue, Connective tissue, Ischemic cardiomyopathy, Cell therapy

## Abstract

**Background:**

Stromal vascular fraction (SVF) can easily be obtained from a mini-lipoaspirate procedure of fat tissue. The SVF contains a mixture of cells including ADSCs and growth factors and has been depleted of the adipocyte (fat cell) population. We evaluated the safety and efficacy of administering SVF intra-myocardially into patients with chronic ischemic cardiomyopathy.

**Methods:**

A total of 28 patients underwent a local tumescent liposuction procedure to remove approximately 60 ml of fat tissue. The fat was separated to isolate the SVF and the cells were delivered into the akinetic myocardial scar region using a transendocardial delivery system (MyoCath^®^) in patients who had experienced a previous myocardial infarct. The subjects were then monitored for adverse events, ejection fraction via echocardiogram and six-minute walk test (6MWT) over a period of 6 months.

**Results:**

The average EF was 29 % at baseline and significantly increased to 35 % at both 3 and 6 months. Patients walked an average of 349 m at baseline and demonstrated a statistically significant improvement at 3 and 6 months’ post treatment of more than 80 m.

**Conclusions:**

Overall, patients were pleased with the treatment results. More importantly, the procedure demonstrated a strong safety profile with no severe adverse events or complications linked to the therapy.

*Trial registration* NCT01502514

Name of registry: http://www.clinicaltrials.gov

URL: https://www.clinicaltrials.gov/ct2/show/NCT01502514?term=adipose+cells+heart&rank=4

Date of registration: December 27, 2011

Date of enrollment: January 2012

## Background

Heart disease is the number one cause of death in the world and the leading cause of death in the United States. Heart disease accounts for one in seven deaths in the U.S., killing over 370,000 Americans per year [1]. The prognosis of patients with heart failure is poor with a 5-year mortality that approaches 50 % [2]. Patients with chronic ischemic cardiomyopathy (IC) have a significantly decreased left ventricular function with ejection fraction of less than 35–40 %. In cases of myocardial infarction, there is an irreversible loss of tissue that cannot be recovered by coronary revascularization due to the fact that the infarcted tissue is not viable [3].

The disease process if IC results includes coronary artery occlusion, which provokes ischemia downstream causing cardiomyocytic apoptosis within minutes. This injury and cell death floods the region with reactive oxygen species and toxic agents that cause the cells around the injury to respond by upregulating and secreting cytokines and chemokines such as tumor necrosis factor alpha (TNF) and a variety of interleukins. The recruitment of pro-inflammatory cells home to the damaged area and attract immune cells. These cells then gradually clear cellular debris and matrix degradation products at the injury site, leaving behind sparse tissue. This gap of tissue later fills with granulation tissue which is mainly composed of blood vessels, macrophages and myofibroblasts. After 1 week, the infarcted area starts to develop into a dense scar with collagen deposits intermingled with myofibroblasts. The ischemic area is rich in inflammatory cytokines and protease activity which harms surrounding healthy cells, further compromising the integrity of the cardiac tissue by causing ventricular dysfunction and electrical instability [4].

Adult mesenchymal stem cells (MSCs) have emerged as a candidate cell type with great potential in regenerative medicine [5, 6]. MSCs are being investigated as a regenerative biologic agent because of their ability to differentiate into multiple tissue types and to self-renew.

The paracrine activity of MSCs is thought to be one of the major means by which these cells mediate anti-inflammatory, anti-apoptotic, anti-fibrotic, angiogenic, mitogenic and wound healing properties. The complex interplay of the biological mediators secreted by MSCs has been shown to be important in regulating regeneration of damaged or diseased organs and tissues of the body. It has also been shown that the pre-curser to the MSC is the pericyte which are the cells present on the microvessels and capillaries throughout the body. These cells become “activated” when an injury is recognized and detach to become medicinal MSCs. An immune-modulatory effect is initiated where other cells are called to help with the healing process while other secreted molecules will establish a regenerative microenvironment by setting up a trophic field [7].

Stem cells derived from a patient’s own fat are referred to as adipose-derived stem cells [8]. Adipose-derived stem cells or ADSCs are multi-potential in that they have the ability to differentiate into a variety of different types of tissue including but not limited to bone, cartilage, muscle, and fat. These cells have also been shown to express a variety of different growth factors and signaling molecules (cytokines), which recruit other stem cells to facilitate repair and healing of the affected tissue. ADSCs are very angiogenic in nature and can promote the growth of new blood vessels. In addition, ADSCs might play a role in the local inflammatory process [9, 10].

A stromal vascular fraction (SVF) can easily be isolated from fat tissue in approximately 30–90 min in a clinic setting using a mini-lipoaspirate technique. The SVF contains a mixture of cells including ADSCs and growth factors and has been depleted of the adipocyte (fat cell) population. It has been shown that cells isolated from the SVF contain an abundance of CD34+ cells [11]. This marker is present on both pericytes and mesenchymal cells. Cells expressing CD34 are also known to reside in a periendothelial location and stabilize endothelial networks. SVF can be used in a point of care setting for a variety of indications and is currently being used in thousands of clinics world-wide with varying degrees of success reported. Adipose tissue is quickly becoming the preferred source for point of care treatments in clinic due to the high number of MSCs that can be obtained and the low number of leukocytes as compared to bone marrow [12]. In addition, adipose tissue has a significantly higher amount of pericytes which are the precursors to MSCs [13, 14].

Stem cells from adipose tissue offer a novel therapy for patients with IC [15, 16]. SVF injected into areas of low perfusion or scars in the cardiac tissue may become populated with angiogenic stem cells, improving blood supply in the area and reducing myocardial scar size [17–19]. SVF is an attractive therapeutic method given that the harvesting process is safe and the cells are readily available in usually large quantities. Transplantation of SVF or ADSCs in animal models of myocardial infarction [20, 21] and dilated cardiomyopathy [22] significantly improves left ventricular cardiac function and decreases mortality after cell transplantation [15, 23–26]. The benefits of ADSCs are postulated to come from their influence on neovascularization of the ischemic tissue and their protection of resident cells [27, 28]. The delivery of angiogenic proteins like angiopoietin1 (Ang1), survival factors like insulin growth factor 1 (IGF-1), and chemokines like stromal cell-derived factor-1 (SDF-1), further enhance the recovery of injured myocardium [29].

Perin et al. reported the results from 21 patients who were injected with SVF via catheter directly into the myocardium. All procedures were well tolerated, safe and feasible. In addition, patients injected with SVF may have preserved ventricular function, myocardial perfusion and exercise capacity [30]. We report the safety and preliminary efficacy results of catheter-based SVF administration in patients with chronic ischemia.

## Methods

### Study design

The open label study was conducted at 2 centers on 28 patients. The protocol was approved by the institutional review board of each institution and all patients provided written informed consent. The trial was funded in part by US Stem Cell Inc., (FKA Bioheart Inc.—Sunrise, FL). SVF was injected intra-myocardially into akinetic tissue under fluoroscopic guidance. Clinical evaluations were scheduled at baseline, 1, 3 and 6 months. The primary safety endpoint was serious adverse events (SAEs) and were defined as any event that was fatal or life-threatening, led to hospitalizations, or required major medical intervention. The primary efficacy endpoint was the change in six-minute walk test (6MWT) distance at 3 and 6 months. The secondary efficacy endpoint was the effect on global left ventricular ejection fraction (LVEF) at 3 and 6 months.

### Patient eligibility

Patients age 18–90 years with a New York Heart Association (NYHA) class II to IV heart failure and LVEF <40 % were eligible for the study. Patients were excluded if they had recent coronary artery bypass graft surgery or cardiac resynchronization therapy (<90 days). Patients with planned revascularization were also excluded. Patients with active cancer or infections including human immunodeficiency virus, hepatitis B or C, or cytomegalovirus were excluded.

### Cell preparation and study intervention

From each patient, approximately 60 ml fat was collected using a 3 mm aspiration cannula with prior administration of tumescent solution. The tissue was prepared using an adipose stromal vascular fraction preparation kit (US Stem Cell, Inc. Sunrise, FL). The adipose tissue was washed with buffered saline and digested using collagenase (Cellase, US Stem Cell, Inc., Sunrise, FL) at 37 °C for 12–30 min with agitation at 5-min intervals. The suspension was centrifuged at 500×*g* for 5 min to collect the SVF as a pellet. The pellet was washed twice and filtered through a 100 μm cell strainer with buffered saline to remove any residual enzyme. The final SVF pellet was resuspended in approximately 4.5ccs of normal saline. Samples were taken to determine the cell quantity and viability.

The SVF was intra-myocardially injected into the targeted treatment region using the MyoCath^®^ (US Stem Cell, Inc. Sunrise, FL) catheter delivery system under fluoroscopic guidance as previously described [31]. Sixteen injections of 0.25ccs each were delivered into the myocardium for a total volume of 4ccs.

### Outcomes

The primary safety outcome was the incidence of SAEs over 6 months. This included death, myocardial infarction, rehospitalization, and arrhythmia. The efficacy outcomes were changes in 6MWT and LVEF by echocardiogram from baseline to 3 and 6 months. Wall thickness was measured in a small subset of patients. This study was designed to primarily assess the safety and feasibility of the percutaneous AdipoCell™ transplantation procedure and secondarily to provide preliminary data regarding the efficacy of intramyocardial SVF transplantation. Formal power calculations were not performed. Two tailed statistical analyses were performed and confidence intervals are presented with 95 % degree of confidence. All statistical tests used a significance level of α ≤0.05. Several patients did not complete some or all of the follow up tests. For those patients, the baseline data was not included in the statistical analysis or the graphs presented.

## Results

### Patient baseline evaluation

A total of 28 patients were enrolled in the study and treated at one of two clinical sites. Baseline clinical characteristics and demographics of the patients are listed in Table 1. Ninety-three percent of the patients were male and 7 % female with an average age of 65.8 (range 29–87). A majority (54 %) of the patients presented as NYHA class III with an average of 28 % LVEF. Twenty-four and eleven patients completed LVEF by echocardiogram at 3 and 6 months, respectively. A small group of four patients completed LVEF at 12 months. Twelve and eight patients completed 6MWT at 3 and 6 months, respectively. Three patients completed 6MWT at 12 months. In addition, three patients completed wall thickness measurements at 1, 3, 6 and 12 months.

**Table 1:** Patient demographics and medical history

Patient parameter	Value
Sex (%)	
Male	93 (26)
Female	7 (2)
Age	
Mean ± SD	65.8 ± 12.0
(Min–max)	29–87
NYHA % (n)	
NYHA II	15 (4)
NYHA III	54 (14)
NYHA IV	31 (8)
Ejection fraction (%)	
Mean ± SD	28.0 ± 0.1
6MWT (m)	
Mean ± SD	349.3 ± 119.8

### Adipose and SVF collection

**Fig. 1:**
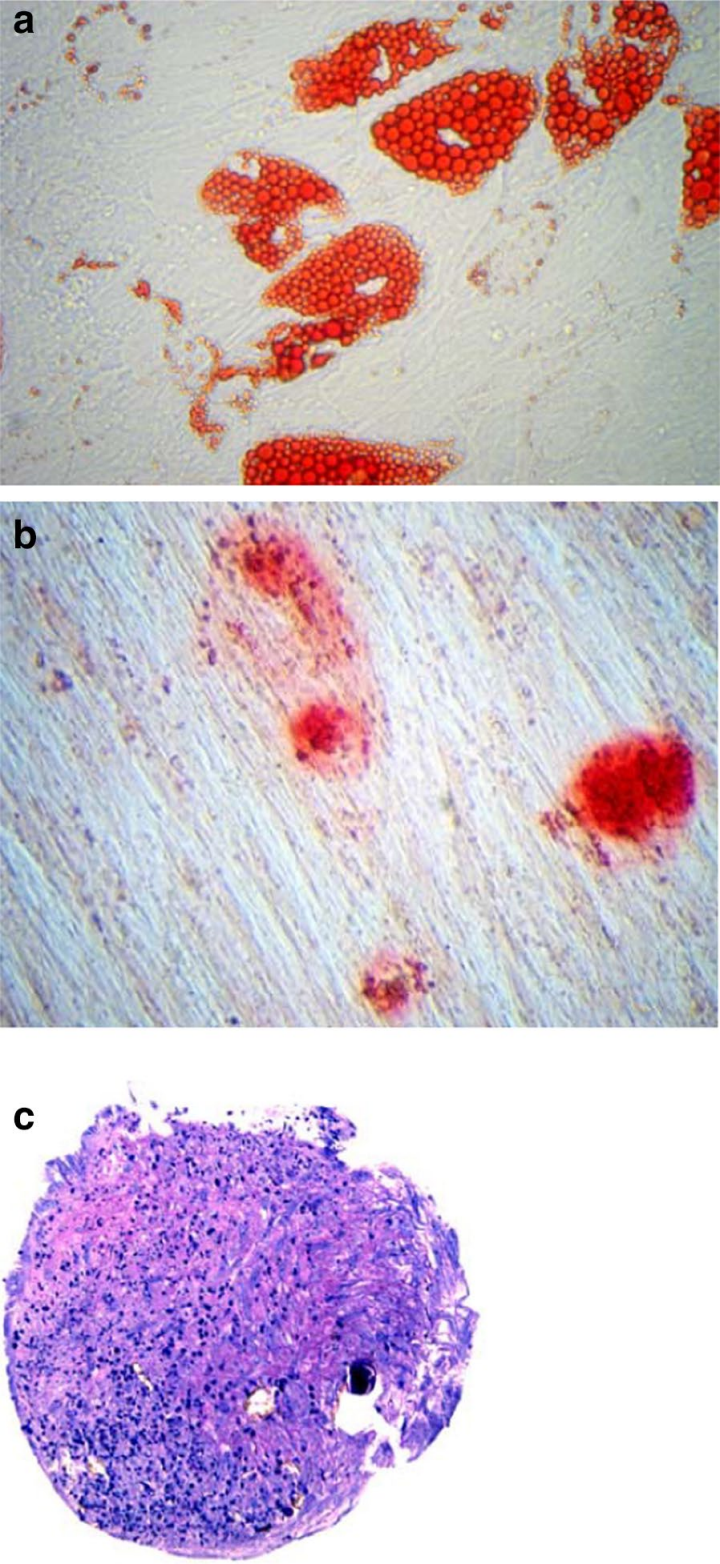
**a** Adipogenesis fat differentiation (*oil red*-O), **b** Osteogenesis bone differentiation (alizarin* red* S), **c** Chondrogenic cartilage differentiation (toluidine blue sodium borate stain)

All procedures were well tolerated and uneventful, resulting in a collection of approximately 60ccs of adipose tissue. Approximately 30–60 million SVF cells were obtained. According to validation studies, the population obtained is at least 50 % positive for CD34 with a viability of greater than 90 % (data not shown). In addition to testing the proliferation capacity of the cells, differentiation assays for adipogenesis (fat), osteogenesis (Bone) and chondrogenesis (cartilage) were completed. The samples shown in Fig. 1 displayed positive differentiation results confirming the presence of multi-potential mesenchymal stem cells.

### Transplantation procedure

The transplantation procedure was successful in 28/28 patients. Patients received approximately 30–60 million cells in 4ccs volume. A total of 16 injections (0.25 mL each) were placed via catheter into the myocardium at half the measured thickness as determined by echocardiography.

### Efficacy outcomes

**Fig. 2:**
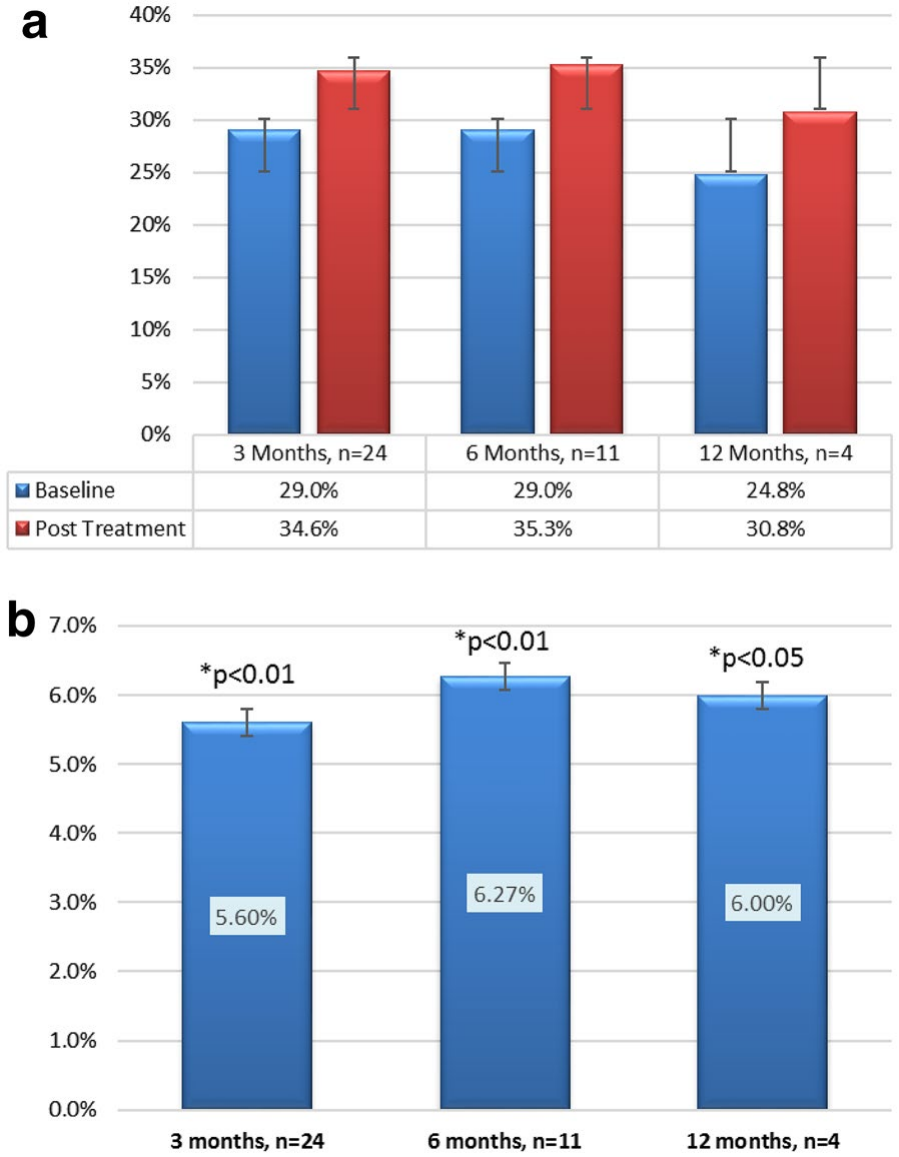
**a** Absolute left ventricular ejection fraction (LVEF), **b** Change in LVEF

The efficacy outcome of the LVEF by echocardiogram demonstrated statistically significant improvement at both 3 and 6 months (Fig. 2). Absolute LVEF went from 29 % at baseline to 35 % at both 3 and 6 months (p < 0.01). Four patients completed 12 month follow up and went from 25 % at baseline to 31 % (p < 0.05). The change in LVEF from baseline was 5.6, 6.3 and 6.0 % at 3, 6 and 12 months, respectively.

**Fig. 3:**
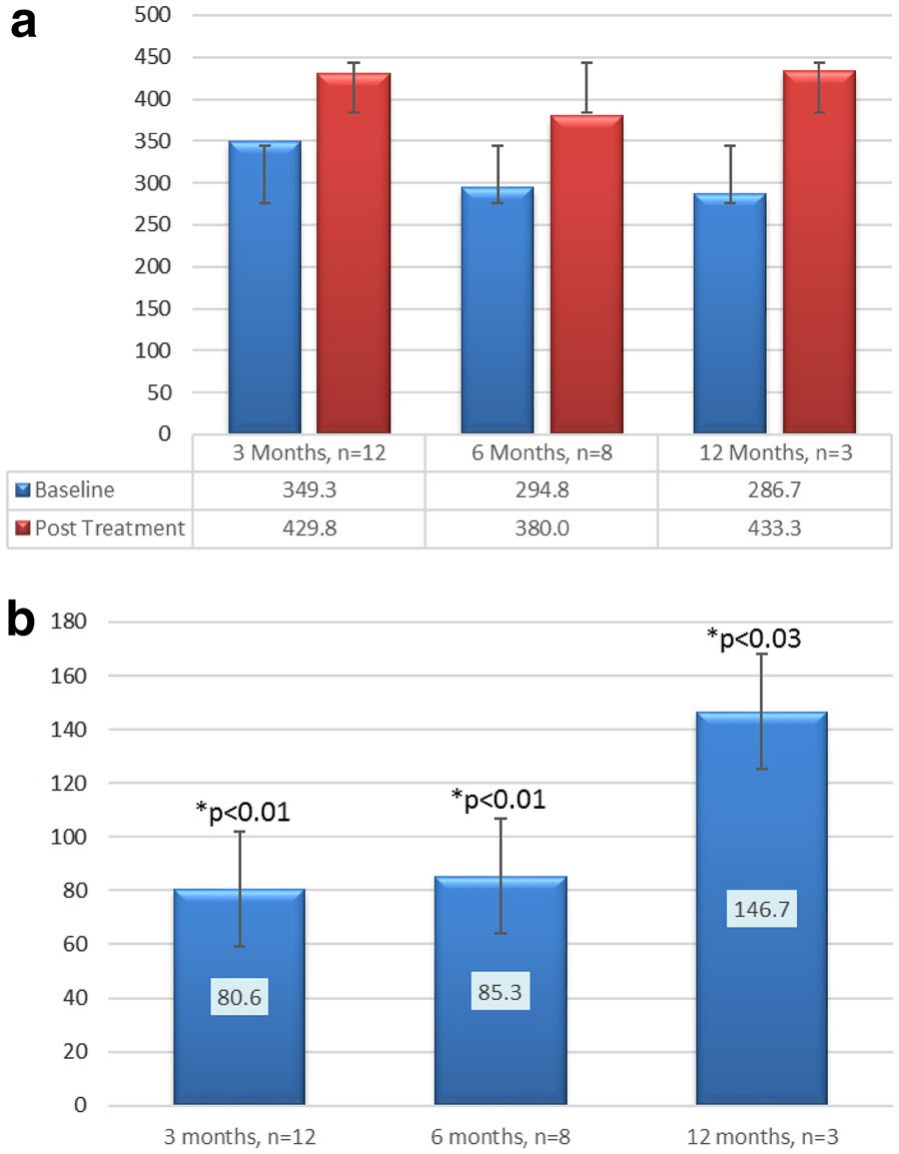
**a** Six minute walk test (6MWT) in m, **b** Change in 6MWT

Patients demonstrated a statistically significant improvement in 6MWT (Fig. 3). Twelve patients went from an average of 349 m at baseline to 430 m at 3 months (p < 0.01). Eight patients went from an average of 295–380 m at 6 months (p < 0.01) and three patients went from 287 to 433 m at 12 months (p < 0.03). This corresponded to an average change of 81, 85 and 147 m at 3, 6 and 12 months respectively.

**Fig. 4:**
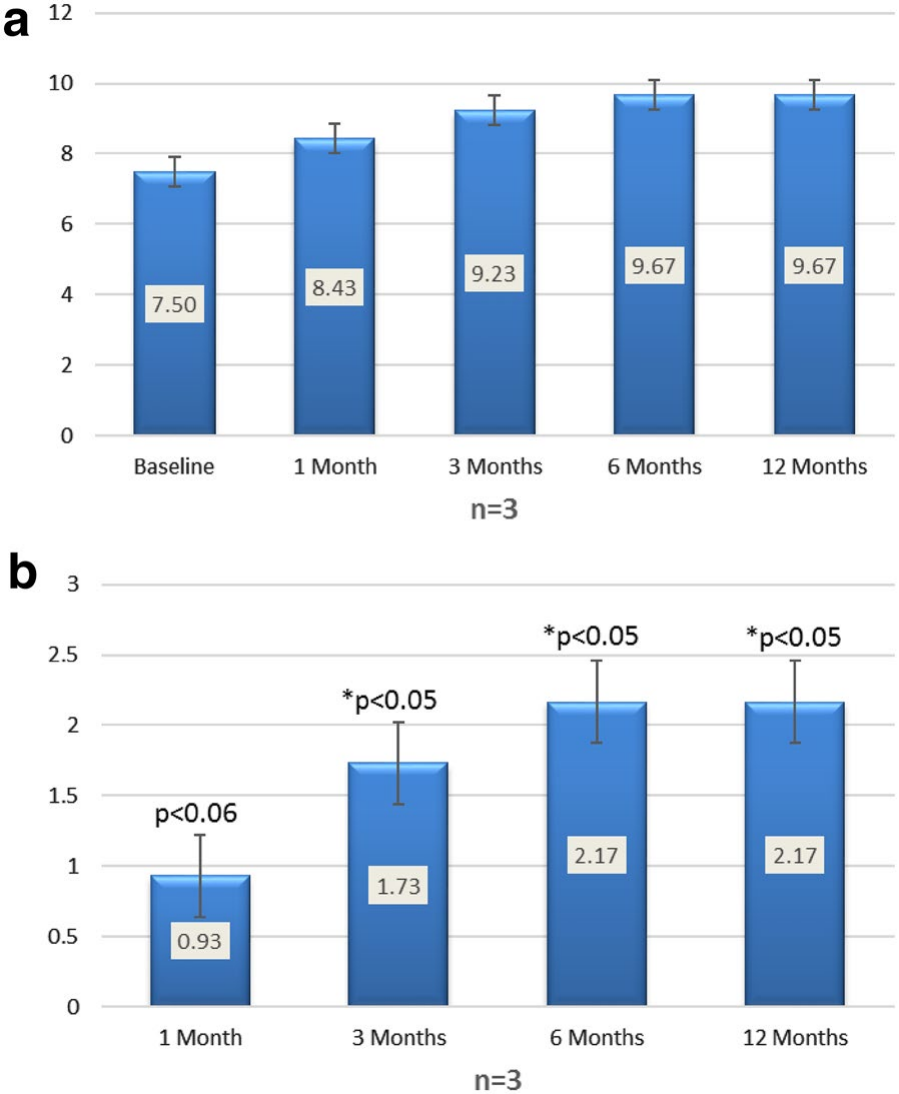
**a** Wall thickness (mm), **b** Change in wall thickness

Wall thickness collected on three patients showed numerical improvement at 1 month and statistical significance at 3, 6 and 12 months (Fig. 4). Patients went from a wall thickness of 7.5 at baseline to 8.4 (p < 0.06), 9.2 (p < 0.05), 9.7 (p < 0.05) and 9.7 (p < 0.05) at 1, 3, 6 and 12 months respectively. This corresponded to an average change of 0.93, 1.73, 2.17 and 2.17.

### Safety analysis

Three patient deaths were reported during a 12 month follow up period. The first patient experienced a bowel obstruction 7 months’ post stem cell procedure. The patient was administered contrast solution to determine the extent of obstruction and went into cardiac arrest. He survived the episode but continued to deteriorate until death. Another patient died 12 months’ post procedure, however, cause of death was not recorded. The patient withdrew from the study at 3 months and did not obtain any follow up tests. The third patient passed 4 weeks post stem cell procedure due to pulmonary thromboembolism. All SAEs were reviewed and adjudicated by the local hospital IRB.

Other reported adverse events included soreness in the abdomen after the mini-liposuction procedure. One patient reported headache and nausea for 48 h post procedure. Patients were instructed to take Tylenol for pain and all events resolved within 7–10 days. One patient experienced a small hematoma at the aspiration site which resolved within 7 days after application of ice. Many patients experienced brief bradycardia or arrhythmia at time of injection which lasted from 30 s to several minutes. All occurrences resolved without intervention.

## Discussion

Chronic heart ischemia is a progressive degenerative disease associated with high rates of death and limited clinical options. In recent years, stem cell therapy has developed with promising preclinical results and preliminary clinical results. Adipose derived stem cells as part of the stromal vascular fraction are a feasible candidate for cardiac indications. The SVF does not require in vitro culture expansion and is easy to collect bedside. These cells can be placed directly into the damaged areas of the myocardium utilizing a minimally invasive catheter technique.

This clinical study demonstrated the safety and feasibility of utilizing the SVF in heart disease patients. No major safety issues were noted and the procedures were well tolerated in all patients. In addition, the rates of death were below those reported for no-option angina patients [32]. We reported three deaths for a total mortality of 10.7 %.

The safety profile is consistent with previously reported results by Perin et al. in the PRECISE trial. This trial showed significant improvements in MVO_2_, total left ventricular mass by MRI, and wall motion score index. In the same trial, LVEF outcomes failed to show significant differences between control and treated patients. This study did not report outcomes on 6MWT or wall thickness. In our study, patients demonstrated a statistically significant improvement in LVEF, 6MWT and wall thickness. Absolute LVEF improved by approximately 6 % at 3, 6 and 12 months post procedure. In addition, patients are walking an additional 80+ m after the stem cell treatment. We believe that exercise capacity or 6MWT may be a better indication of the overall well-being of the patients. Subjective quality of life parameters were reported but these would need to be substantiated in double blind placebo controlled studies.

Although this study suggests that the use of SVF is safe and feasible, the general under powering of the study coupled with the lack of placebo control would render additional studies necessary to determine the true clinical effect of the treatment. In addition, several patients were lost to follow up which could compound the data and create patient bias. Given the encouraging results on this small sample size with statistical significance, large appropriately powered clinical studies blinded to both clinical staff and patients are warranted.

## Conclusions

The current study sought to define the safety and feasibility of percutaneous intramyocardial transplantation of autologous SVF in patients with chronic ischemia cardiomyopathy. Several parameters demonstrated statistically significant improvements over a 6–12 months time period. A true evaluation of efficacy and safety would require larger phase II/III studies. However, the current study does provide encouraging feasibility data regarding the endomyocardial stem cell treatment and suggests some clinical benefit of the SVF therapy in heart failure patients.

## Abbreviations

SVF: stromal vascular fraction
6MWT: six minute walk test
LVEF: left ventricular ejection fraction
ADSCs: adipose derived stem/stromal cells
IC: ischemic cardiomyopathy
MSC: mesenchymal stem cell
SAE: severe adverse event
NYHA: New York Heart Association
